# Bimodular effects of D614G mutation on the spike glycoprotein of SARS-CoV-2 enhance protein processing, membrane fusion, and viral infectivity

**DOI:** 10.1038/s41392-020-00392-4

**Published:** 2020-11-17

**Authors:** Xiaoyi Jiang, Zhengrong Zhang, Chenxi Wang, Hongguang Ren, Lihua Gao, Haoran Peng, Zubiao Niu, He Ren, Hongyan Huang, Qiang Sun

**Affiliations:** 1 Beijing Institute of Biotechnology, 20 Dongda Street, Beijing, 100071 China; 2 Department of Oncology, Beijing Shijitan Hospital of Capital Medical University, 10 TIEYI Road, Beijing, 100038 China; 3 Department of Microbiology, Second Military Medical University, Shanghai, 200433 China

**Keywords:** Infectious diseases, Microbiology

**Dear Editor,**

By the end of June 2020, the pandemic of coronavirus diseases 2019 (COVID-19) had resulted in more than 10 million individuals, all over the world, being infected with the severe acute respiratory syndrome coronavirus 2 (SARS-CoV-2).^1^ The high contagiousness of SARS-CoV-2 virus was largely attributed to the relatively unique sequence composition in its spike (S) glycoprotein, which is in charge of the host entry by interacting with its cellular receptor angiotensin-converting enzyme 2 (ACE2).^2,3^ The S glycoprotein can be processed into an N terminal S1 fragment that is responsible for receptor binding, and a C terminal S2 fragment that functions to promote membrane fusion.^4^ SARS-CoV-2 is a type of single-stranded positive-sense ribonucleic acid (RNA) virus, and an increasing number of mutations were identified across the SARS-CoV-2 genome, including the region encoding S glycoprotein. Nevertheless, the functional implications of these mutations remain largely unknown, we here reported the mutation analysis of S gene and a functional exploration of the dominant D614G mutation.

In total, 9002 S gene sequences were extracted from the high-quality complete genomes deposited in the GISAID EpiCoV database as of April 28, 2020. Sequence alignment identified 6253 non-synonymous mutations at 82 different sites of the S gene (Supplementary Table S1 and Fig. 1a). While the majority of the mutations (for 81 sites) were in low frequency (<0.6%), the D614G stood out as a prominent mutation, accounting for about 90% (5583/6253) mutations identified (Supplementary Table S1), with a site-specific mutation frequency of >62% (5583/9002) (Fig. 1a), suggesting potential impacts on the function of S protein.

**Fig. 1:**
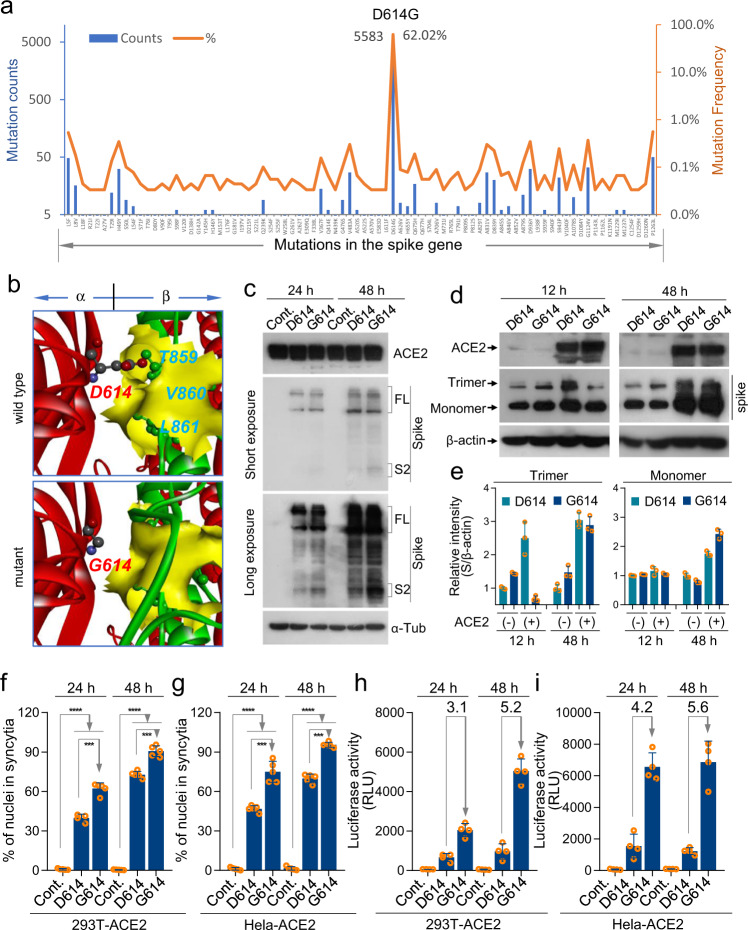
Enhanced infectivity of D614G mutant of SARS-CoV-2 virus. **a** The mutation profile of SARS-CoV-2 spike glycoprotein. From a set of 9002 SARS-CoV-2 genome sequences, mutations were identified at 82 positions across the gene encoding S glycoprotein with the D614G being the dominant mutation (>62%); for the plot of mutation counts (the left *Y* axis), only those showing more than 5 counts were displayed. **b** Three-dimensional structure modeling of the D614 (upper) and G614 (lower) S glycoprotein colored by chain. The α subunit in red is on the left, the neighboring β subunit in green is on the right. The D614, G614, and T859 residues are displayed in the style of scaled ball and stick with simulated surfaces in yellow. **c** Detection of the expression of the indicated proteins by Western blot. The spike protein was blotted by an antibody against the S2 region. **d**, **e** Altered expression of spike trimer in G614 mutant. **d** Expression of two spike proteins in the absence and presence of ACE2 in 293T cells at the time points of 12 and 48-h post transfection by Western blot. **e** Quantification of the relative expression of spike trimer and monomer with the respective D614 expression in the absence of ACE2 as the reference. All results were normalized by the expression of β-actin. Data are mean ± SD of triple quantification. **f**, **g** Quantification of syncytia formation upon expression of the indicated S glycoprotein in 293T-ACE2 cells (**f**) and Hela-ACE2 cells (**g**). Data are the mean ± SD of results from 4–5 fields (20x objective lens). ***p < 0.001; ****p < 0.0001. More than three replicates were done for the experiment. **h**, **i** The expression of the luciferase reporter in 293T-ACE2 cells (**h**) and Hela-ACE2 cells (**i**) upon infection of viruses pseudotyped with D614 or G614 S glycoproteins as indicated. Data are the mean ± SD of the results of quadruplicate. Fold changes between D614 and G614 genotypes are displayed. More than three replicates were done for the experiment

To explore the potential effects of the D614G mutation on the spatial structure of S glycoprotein, we performed three-dimensional (3D) modeling with the template of published SARS-CoV-2 S structure (6vxx.pdb) by the Modeling algorithm at SWISS-MODEL. The native S protein trimer was comprised of 3 identical subunits, the Aspartic acid residue at position 614 (D614) of wild type S protein located at a region interfacing with the neighboring subunit (Fig. 1b, left α subunit in red), and was spatially close to and potentially interacted with the T859 residue of neighboring subunit by an inter-subunit hydrogen bond (Fig. 1b, left β subunit in green). This potential inter-subunit interaction mediated by D614-T859 was disrupted by the D to G mutation due to the loss of the side chain, which eliminated the inter-subunit hydrogen bond and may increase the flexibility of the main chain, as indicated by the surface simulation of T859, V860, L861 residues of the neighboring subunit (Fig. 1b, left β subunit in yellow). Meanwhile, the backbone amine of the glycine (G) residue shortened the hydrogen bond with the carboxyl group of neighboring amino acids (A647) within the same subunit and strengthened it, which might locally stabilize the S protein. Thus, the D614G mutation may conceivably affect the stability of the S protein trimer and its related functions, such as host entry and viral infectivity.

In order to examine the potential functional outcomes of the D614G mutation, we made two constructs expressing the wild type (D614) and mutant (G614) SARS-CoV-2 S glycoprotein, respectively. Transfection of the same amount of these two constructs into 293T cells expressing ACE2 (293T-ACE2) effectively expressed full length (FL) S protein (Fig. 1c) at both 24 and 48-h post-transfection as detected by an antibody against the S2 region. Whereas, increased S2 production, which is resulted from protease-mediated cleavage of S protein, was observed in the G614 mutant as compared with that in the wild type D614 transfectant, the difference was even more obvious for transfectants of 48-h post-transfection (Fig. 1c), suggesting that the D614G mutation enhanced S protein processing, which is consistent with the idea that the D614G mutation may affect the stability of the S protein trimer as suggested above based on structural analysis (Fig. 1b). Interestingly, the expression of the super-shifted band above the full length S protein, representing for the trimer, was higher in G614 than D614 in the absence of ACE2 at both 12 and 48-h post-transfection, but was lower in G614 than D614 in the presence of ACE2 at 12-h post-transfection, and the difference disappeared at 48-h post-transfection, probably due to the saturated effects from ACE2 which could clearly promote the accumulation of overexpressed S protein (Fig. 1d, e). The results fit well with a bimodular effect of D614G mutation on S-trimer stability depending on ACE2 engagement, i.e., G614 mutation increased S-trimer stability on virion in the absence of ACE2, but promoted S-trimer dissociation, upon engaged with ACE2, to expose the S2 fragment (Fig. 1c), which conceivably contributed to high contagiousness.

Since SARS-CoV-2 infection could induce membrane fusion (Supplementary Fig. S1) via S expression, the increased S2 production may predict the enhanced ability of the D614G mutant in inducing membrane fusion. To test this hypothesis, we took the advantages of syncytium formation assay as a surrogate of S protein-induced membrane fusion. As shown in Supplementary Fig. S2, a number of syncytia were formed in 293T-ACE2 cells 24-h post-transfection of wild type D614 S construct, while the G614 mutant exhibited significantly stronger induction of syncytium formation as indicated by higher fusion index calculated as the percentage of nuclei in syncytia (Fig. 1f), the difference maintained at 48-h post-transfection, when extensive syncytium formation had occurred (Supplementary Fig. S2 and 1f). the effect of D614G on syncytium formation was also confirmed in Hela cells engineered to stably express ACE2 cells (Fig. 1g). These results indicate that the D614G mutation may endow enhanced fusion ability to the SARS-CoV-2 S glycoprotein.

To examine whether the enhanced fusion ability could be translated into enhanced virus infection, we made viruses pseudotyped with wild type and G614 S proteins, and infected 293T-ACE2 cells and Hela-ACE2 cells with the same amount of virus particles. As shown in Fig. 1h, i, the viruses pseudotyped with G614 mutant displayed luciferase activities about 3.1 ~ 4.2 times higher than those pseudotyped with wild type (D614) S protein by the time of 24-h post infection; and the fold changes increased to 5.2 ~ 5.6 by the time of 48-h post infection. Moreover, the luciferase activities positively correlated with membrane fusion indexes (Fig. 1f–i). Thus, these data are consistent with the notion that the D614G mutation enhanced SARS-CoV-2 infectivity via membrane fusion-mediated host entry.

Interestingly, the Hela-ACE2 cells, but not 293T-ACE2 cells, expressed considerable amounts of TMPRSS2, which was also involved in the phenotypes mediated by S-G614 as the treatment of Camostat, a TMPRSS2 inhibitor, compromised S-G614-induced phenotypes (Supplementary Fig. S3).

Consistent with our finding, a recent study by Kober et al.^5^ reported that the SARS-CoV-2 variant containing the D614G mutation, initially beginning spreading in Europe, rapidly became a dominant form in regions where it was introduced. And the recurrent pattern of the increase occurred at multiple geographic levels, indicating high efficacy of transmission. Together with our findings, it is proposed that the SARS-CoV-2 variant of G614 genotype is highly transmissible, which is probably attributed to an increased S2 production associated with bi-modularly altered S-trimer stability, leading to enhanced membrane fusion and host entry. Therefore, particular attention should be paid to the emergence of this variant in a region to help prevent recurrent COVID-19 outbreak.

## Supplementary information

Supplemenary files
