# Supplementary material for: Bimodular effects of D614G mutation on the spike glycoprotein of SARS-CoV-2 enhance protein processing, membrane fusion, and viral infectivity
Source: Signal Transduct Target Ther. 2020 Nov 17;5:268. doi: 10.1038/s41392-020-00392-4 (PMC7670837; doi:10.1038/s41392-020-00392-4)
Supplement: Supplementary file 1 — Supplemenary files [file 41392_2020_392_MOESM1_ESM.docx]

Supplementary Materials for

Bimodular effects of D614G mutation on the spike glycoprotein of SARS-CoV-2 enhance protein processing, membrane fusion and viral infectivity

Xiaoyi Jiang^1,2†^, Zhengrong Zhang^1†^, Chenxi Wang^1†^, Hongguang Ren^1†^, Lihua Gao^1†^, Haoran Peng^3^, Zubiao Niu^1^, He Ren^1,2^, Hongyan Huang^2*^, Qiang Sun^1*^

† Equal contribution

* To whom correspondence should be addressed.

Qiang Sun

Email: sunq@bmi.ac.cn.

Hongyan Huang

Email: hhongy1999@126.com

**This PDF file includes:**

Materials and Methods

Figures. S1 to S3

Tables S1 to S5

Materials and Methods

**Bioinformatics**

For mutation analysis, genome sequences were downloaded from GISAID EpiCoV database as of April 28, 2020. Only complete sequences with high coverage and > 95% of non-ambiguous sites were included, resulting a data set of 9002 genomes. The spike gene sequences were extracted from each of the genome. The alignment was done with MAFTT v7.455. The alignment was manually curated to remove potential artifacts. The relative substitution diversity of each position of genome was calculated by

$$diversity_{pos}=1-\sum_{nt} \left(\frac{N_{nt}^{pos}}{N_{Strains}}\right)^{2}$$

where N_nt_^pos^ represents the number of occurrences of nucleotide (nt) in position (pos), and N_strain_ is the total number of genomes used in this analysis.

The 3D structure modeling of SARS-CoV-2 S glycoprotein was performed by the Modelling algorithm at SWISS-MODEL (https://swissmodel.expasy.org/) with the template of 6vxx.pdb from RSCB protein data bank (http://www.rcsb.org/).

**Cell culture**

The 293T, 293T-ACE2 and Hela-ACE2 cells were maintained in DMEM (MACGENE Tech Ltd., Beijing, China) supplemented with 10% fetal bovine serum (Kang Yuan Biol, Tianjin, China) and 1% Penicillin-Streptomycin (MACGENE Tech Ltd., Beijing, China). All cells were incubated with 5% CO2 at 37°C.

**Constructs**

The codon-optimized SARS-CoV-2 S cDNA was synthesized at Genscript Biotech Corporation (Nanjing, China). The wild type S genes of SARS-CoV-2 and the D614G mutation were cloned into pSecTag2-Hygro-A through seamless homologous recombination. Please find in supplementary table for detail information on the constructs used in this study.

**Cell fusion**

For cell fusion assay, about 6.5 × 10^5^ cells were plated per well in 6-well plate precoated with type I collagen (354236, BD Bioscience) and cultured for 24 h. Cells were then transfected with respective constructs by Lipofectamine LTX and Plus Reagent (Invitrogen, 1784283, USA) following the protocol provided. Images of 5 fields (20x objective lens) were taken on Hoechst-stained cells 24 h post transfection by Nikon microscope. Nucleus counting was performed by NIS elements AR software (Nikon, Japan). The fusion index (FI) was calculated as “% of nuclei in fused cells”.

**Western blotting**

Cells were lysed on ice with cold Radio-Immunoprecipitation Assay (RIPA) buffer containing phosphatase-protease inhibitors (CWBiotech, Beijing) for 20 min followed by ultrasound (power 40%, work 6 s, stop 9 s, 5 times in total). After being centrifuged at 12,000 rpm for 10 min, the supernatant was collected for SDS-PAGE electrophoresis followed by transferring onto the Polyvinylidene Fluoride (PVDF) membrane (0.2 μm, Millipore). The PVDF membrane, blocked with 5% skimmed milk for 1 h at room temperature, was then blotted with primary antibodies in 5% BSA for 12 h at 4°C or 4 h at room temperature, followed by one-hour secondary antibodies at room temperature. The primary antibodies used: ACE2 (Proteintech, 1:3000, 66699-1-Ig), SARS-CoV-2 spike (GeneTex, 1:2000, GTX632604), TMPRSS2 (Proteintech, 1:1000, 14437-1-AP), α-Tubulin (Proteintech, 1:1000, 11224-1-AP), b-Actin (Proteintech, 1:8000, 60008-I-Ig). The secondary antibodies used: anti-rabbit IgG HRP (CST, 1:3000, #7074), anti-mouse IgG HRP (CST, 1:3000, #7076).

**Pseudovirus production**

The mouse sarcoma virus（MSV）based SARS-CoV-2 S, and SARS-CoV-2 D614G pseudotypes were prepared as previously described. HEK293T cells were co-transfected with an S encoding-plasmid, a Gag-Pol packaging construct (Addgene, 8449, USA) and the pQCXIP retroviral vector (Clontech, USA) expressing a luciferase reporter by using Lipofectamine LTX and Plus Reagent (Invitrogen, 1784283, USA) according to the manufacturer’s instructions. Cells were incubated for 6 h at 37°C with transfection medium. Then transfection medium was changed with DMEM containing 10% Fetal Bovine Serum (FBS) was added for 48 h. The supernatants were then harvested and filtered through 0.45 μm membranes and then frozen at -80°C.

**Pseudovirus titration**

The titers of the pseudoviruses were calculated by determining the number of viral RNA genomes per mL of viral stock solution using RT-qPCR with primers that target LTR. Briefly, viral RNAs were isolated using TRIzol (Invitrogen, #15596026). One microgram of total RNA was converted into cDNA using TransScript^®^ One-Step gDNA Removal and cDNA Synthesis SuperMix (Transgen Biotech, #AT311-02) according to manufacturer’s instruction. The quantitative PCR (qPCR) was performed on 15 ng of cDNA from each sample using SYBR Green Real-time PCR Master Mix (TOYOBO, #QPK-201) based on the recommendations of manufacturer. pQCXIP-luciferase-vector was used to generate standard curves. The S-D614 and S-G614 protein pseudotyped viruses were adjusted to the same titer (copies/mL or CCID50/mL) for the following experiments. Primers pairs spanning at least two exons were confirmed by NCBI. Sense primer: 5′-ATTCCCAATAAAGCCTCT-3′, anti-sense primer: 5′-GGTAGTCAATCACTCAGA-3′.

**Pseudovirus assay**

293T-ACE2 cells and Hela-ACE2 cells were cultured in DMEM medium supplemented with 10% FBS and 1% PenStrep. 293T-ACE2 cells and Hela-ACE2 cells were plated into 96 well plates at a density of 0.5×104 per well for 16 h. About 1.15 × 10^4^ copies of virus in the volume of 50 μL and 50 μL DMEM was added to the wells. After 12 h, 100 μL 10% FBS and 1% PenStrep containing DMEM was added to the cells for 24 h and 48 h. Following the 24 h and 48 h infection, 100 μL One-Glo-EX (Promega, E6120) was added to the cells in equivalent culturing volume and incubated in the dark for 10 min prior to reading on an Enspire 2300 multilable reader (Perkin Elmer, USA). Measurements were done at least in triplicate and relative luciferase units (RLU) were plotted.

**Statistics**

Data were expressed as means with standard deviations (SD). P-values were calculated using two-tailed Student's t-test from GraphPad Prism software, and P-values less than 0.05 were considered statistically signiﬁcant

**Supplemental Figures**


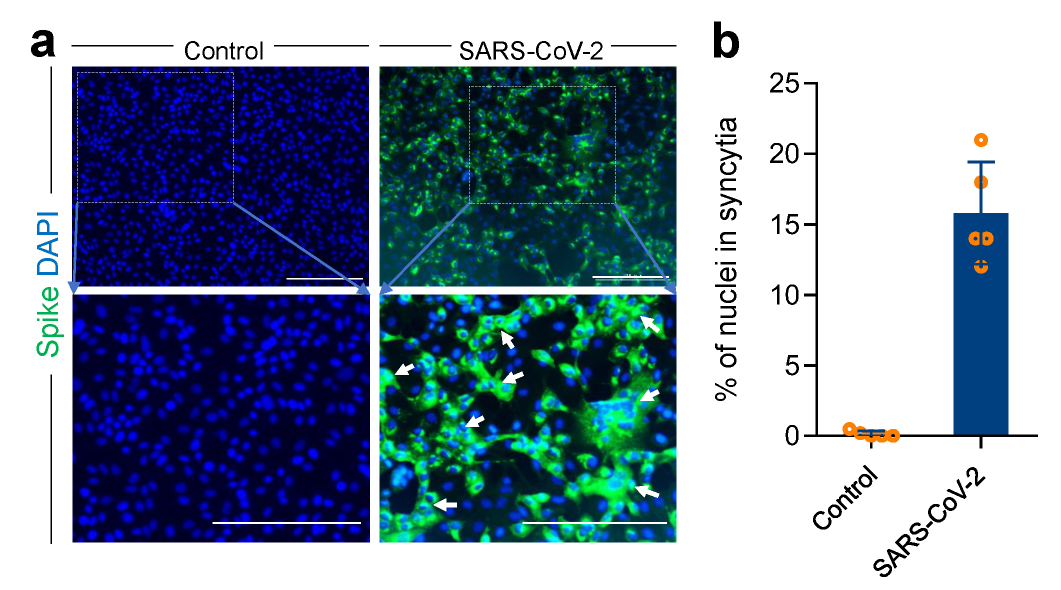


Figure. S1.

**Syncytia formation in Vero-ACE2 cells infected with SARS-CoV-2 authentic viruses**. (a) Representative images for syncytia formation in control and SARS-CoV-2 infected Vero-ACE2 cells. Cells were stained with anti-SARS-CoV-2 S antibody in green. Control: no infection. Scale bars: 200 μm. Arrows indicate syncytia. Note: not all the cells were infected and positive in spike staining. (b) Quantification of syncytia formation in the control cells and the spike-positive cells (SARS-CoV-2). Data are the mean ± SD from 5 fields.


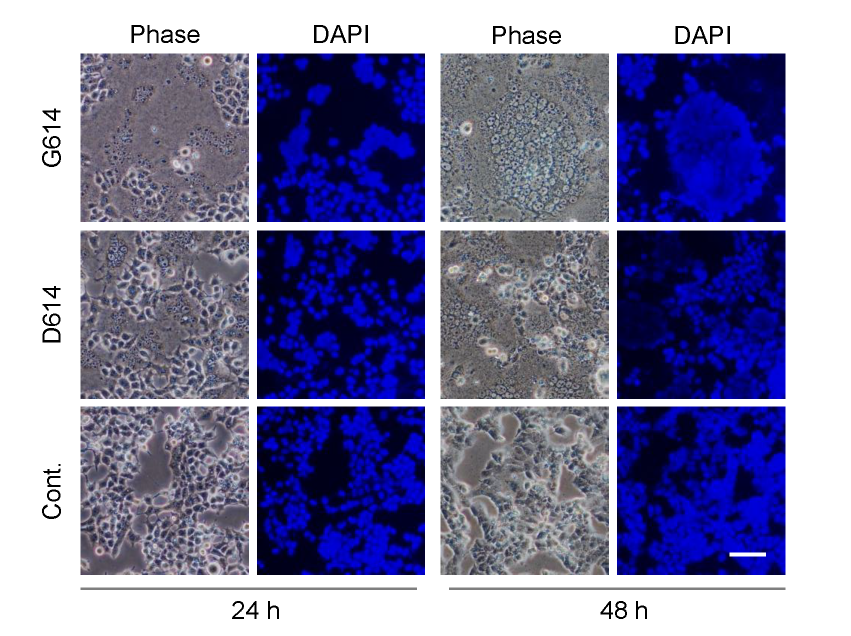


Figure. S2.

**Protein induced syncytia formation in 293T-ACE2 cells**. Representative images for cell fusion upon expression of the indicated S glycoprotein in 293T-ACE2 cells. Scale bars: 50 μm.


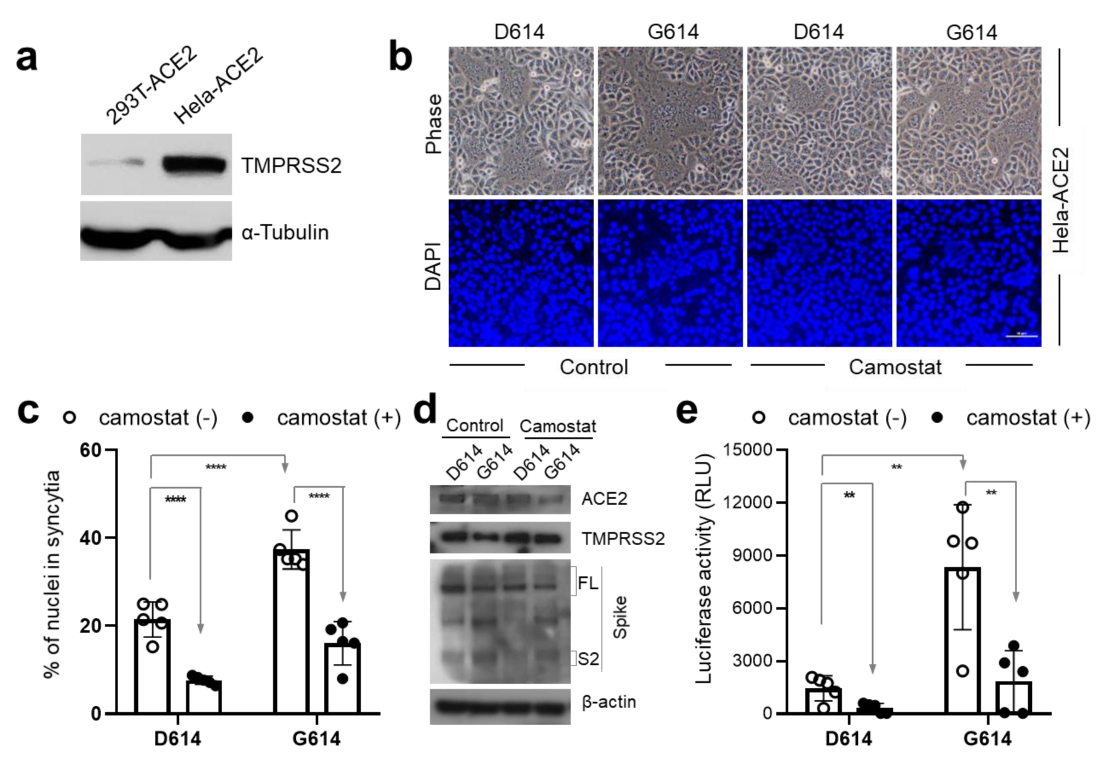
**Figure. S3.**

**The effects of SARS-CoV-2 S protein could be inhibited by Camostat in Hela- ACE2 cells that express TMPRSS2**. (a) Expression of TMPRSS2 detected by Western blot. (b) Representative images for cell fusion upon expression of the indicated S glycoprotein in Hela-ACE2 cells. Scale bars: 50 μm. (c) Quantification of syncytia formation upon expression the indicated S glycoprotein in Hela-ACE2 cells. Data are the mean ± SD of results from 5 fields (20x objective lens). ****: p < 0.0001. Note: to avoid over-fusion caused by G614 in Hela-ACE2 cells, quantification was performed 12 hours post transfection. (d) Detection of the expression of the indicated proteins by Western blot. The spike protein was blotted by an antibody against the S2 region. (e) The expression of luciferase reporter in Hela-ACE2 cells upon infection of viruses pseudotyped with D614 or G614 S glycoproteins as indicated. Data are the mean ± SD of results of five replicates. **: p < 0.01. Camostat was treated at the final concentration of 300 μM.Type or paste caption here. Create a page break and paste in the Figure above the caption.

Table S1.

Mutation profile in the spike gene of SARS-CoV-2.

| **Mutation** | **Counts** | **%** |
| --- | --- | --- |
| **D614G** | **5583** | 62.02% |
| **P1263L** | **50** | 0.56% |
| **L5F** | **48** | 0.53% |
| **G1124V** | **33** | 0.37% |
| **H49Y** | **31** | 0.34% |
| **D936Y** | **31** | 0.34% |
| **V483A** | **27** | 0.30% |
| **A831V** | **27** | 0.30% |
| **S943P** | **22** | 0.24% |
| **D839Y** | **20** | 0.22% |
| **Q675H** | **17** | 0.19% |
| **L8V** | **16** | 0.18% |
| **V367F** | **14** | 0.16% |
| **T29I** | **12** | 0.13% |
| **A879S** | **11** | 0.12% |
| **A1078S** | **10** | 0.11% |
| **S50L** | **9** | 0.10% |
| **Q239K** | **9** | 0.10% |
| **G476S** | **9** | 0.10% |
| **A626V** | **8** | 0.09% |
| **A706V** | **8** | 0.09% |
| **A829T** | **8** | 0.09% |
| **L54F** | **7** | 0.08% |
| **H655Y** | **7** | 0.08% |
| **T791I** | **7** | 0.08% |
| **A846V** | **7** | 0.08% |
| **S98F** | **6** | 0.07% |
| **Y145H** | **6** | 0.07% |
| **H146Y** | **6** | 0.07% |
| **Q414E** | **6** | 0.07% |
| **A520S** | **6** | 0.07% |
| **A845S** | **6** | 0.07% |
| **S939F** | **6** | 0.07% |
| **M1229I** | **6** | 0.07% |
| **M1237I** | **6** | 0.07% |
| **L176F** | **5** | 0.06% |
| **S254F** | **5** | 0.06% |
| **S255F** | **5** | 0.06% |
| **G261V** | **5** | 0.06% |
| **Q677H** | **5** | 0.06% |
| **S704L** | **5** | 0.06% |
| **Mutation** | **Counts** | **%** |
| **R765L** | **5** | 0.06% |
| **P1162L** | **5** | 0.06% |
| **L18F** | **4** | 0.04% |
| **S71F** | **4** | 0.04% |
| **V90F** | **4** | 0.04% |
| **D138H** | **4** | 0.04% |
| **G181V** | **4** | 0.04% |
| **I197V** | **4** | 0.04% |
| **S221L** | **4** | 0.04% |
| **A262T** | **4** | 0.04% |
| **A522S** | **4** | 0.04% |
| **P812S** | **4** | 0.04% |
| **D1260N** | **4** | 0.04% |
| **R21I** | **3** | 0.03% |
| **T22I** | **3** | 0.03% |
| **A27V** | **3** | 0.03% |
| **T76I** | **3** | 0.03% |
| **D80Y** | **3** | 0.03% |
| **T95I** | **3** | 0.03% |
| **V120I** | **3** | 0.03% |
| **G142A** | **3** | 0.03% |
| **M153T** | **3** | 0.03% |
| **D215Y** | **3** | 0.03% |
| **W258L** | **3** | 0.03% |
| **E309Q** | **3** | 0.03% |
| **F338L** | **3** | 0.03% |
| **N439K** | **3** | 0.03% |
| **A570V** | **3** | 0.03% |
| **E583D** | **3** | 0.03% |
| **L611F** | **3** | 0.03% |
| **M731I** | **3** | 0.03% |
| **P809S** | **3** | 0.03% |
| **A852V** | **3** | 0.03% |
| **L938F** | **3** | 0.03% |
| **S940F** | **3** | 0.03% |
| **V1040F** | **3** | 0.03% |
| **D1084Y** | **3** | 0.03% |
| **P1143L** | **3** | 0.03% |
| **K1191N** | **3** | 0.03% |
| **C1254F** | **3** | 0.03% |
| **D1259H** | **3** | 0.03% |

Table S2.

Information for constructs used in this study.

|  | Plasmids | Construct Method | Backbone | Site | DNA | Primer | Primer Sequence (5'→3') |  |
| --- | --- | --- | --- | --- | --- | --- | --- | --- |
| 1 | pSecTag2-COV2-S | Homologous Recombination | pSecTag2 Hygro A | *Xho* I | COV2-S | SARI-F1 | AGCTTGGTACCGAGCTCGCAGTGCGTCAATCTGACAACTCG |  |
|  |  |  |  |  |  |  |  |  |
|  |  |  |  | *Bam*H I |  | SARI-R1 | TTCGGGCCCTCCTCGAGCGGTGTAATGCAGCTTCACGC |  |
|  |  |  |  |  |  |  |  |  |
| 2 | pSecTag2-COV2-D614G | Homologous Recombination | pSecTag2 Hygro A | *Xho* I | COV2-R683A-1 | HA-S1-F | ACGAAGCTTGGTACCGAGCTCG |  |
|  |  |  |  |  |  | D614G-R | GTACAATTCACGCCCTGATACAGCACGGCCACCTG |  |
|  |  |  |  | *Bam*H I | COV2-R683A-2 | D614G-F | TGTATCAGGGCGTGAATTGTACCGAGGTGCC |  |
|  |  |  |  |  |  | S2-R | TGAGTTTTTGTTCGGGCCCTCCTC |  |
| 3 | pQCXIP-EGFP-Luciferase | Cohesive End Ligation | PNL4.3.luc.R-E | *Eco*R V | Luciferase | Luc-F1 | ACGGTACCGCGGGCCACCATGGGTGGCGCGGCCGC |  |
|  |  |  |  |  |  |  |  |  |
|  |  |  |  | *Bam*H I |  | Luc-R1 | AGAGCCTGGACCACTGATCTAGGTCTCGAGCAATTTGGACTTTCCGCCC |  |
|  |  |  |  |  |  |  |  |  |

Table S3.

Constructs from Addgene.

| Name | Source | Cat. No | Inserts | Purpose |
| --- | --- | --- | --- | --- |
| pCMV-VSV-G | Addgene | 8454 | VSV-G | Envelope protein for producing lentiviral and MuLV retroviral particles |
| pUMVC | Addgene | 8449 | gag-pol | Packaging plasmid for producing MuLV retroviral particles. |

Table S4.

Antibodies used in this study.

| Antibody | Company | Cat. No | Source | Type | Dilution |
| --- | --- | --- | --- | --- | --- |
| α-Tubulin | proteintech | 11224-1-AP | Rabbit | Polyclonal | WB 1:1000 |
| ACE2 | proteintech | 66699-1-Ig | Mouse | Monoclonal | WB 1:3000 |
| Spike antibody [1A9] | Genetex | GTX632604 | Mouse | Monoclonal | WB 1:2000 |
| Anti-rabbit IgG HRP | CST | 7074 | Goat |  | WB 1:3000 |
| Anti-mouse IgG HRP | CST | 7076 | Horse |  | WB 1:3000 |
| TMPRSS2 | proteintech | 14437-1-AP | Rabbit | Polyclonal | WB 1:1000 |
| beta-actin | proteintech | 60008-I-Ig | Mouse | Monoclonal | WB 1:8000 |

Table S5.

Cells used in this study.

| Cell lines | Source | Tissue Source | Cell type | Growth Properties | Culture medium |
| --- | --- | --- | --- | --- | --- |
| HEK 293T | maintained in the lab | human, kidney | epithelial cell | adherent | DMEM+10% FBS+1% Penicillin-Streptomycin |
| HEK-293T-ACE2 | constructed in the lab | human, kidney | epithelial cell | adherent | DMEM+10% FBS+1% Penicillin-Streptomycin |
| Hela-ACE2 | a gift from Dr. Hui Zhong at Beijing Institute of Biotechnology | human,cervix | epithelial cell | adherent | DMEM+10% FBS+1% Penicillin-Streptomycin |
